# Changes in health-related quality of life and the associated factors among Myanmar migrants with tuberculosis: a cohort study

**DOI:** 10.1186/s12879-021-06070-2

**Published:** 2021-04-21

**Authors:** Myo Minn Oo, Naris Boonathapat, Htet Ko Ko Aung, Petchawan Pungrassami, Tippawan Liabsuetrakul

**Affiliations:** 1 Epidemiology Unit, Faculty of Medicine, Prince of Songkla University, Hat Yai, Songkhla, Thailand; 2 Mae Sot General Hospital, Mae Sot, Tak, Thailand; 3 Shoklo Malaria Research Unit, Mahidol-Oxford Tropical Medicine Research Unit, Faculty of Tropical Medicine, Mahidol University, Bangkok, Thailand; 4 Bureau of Tuberculosis, Ministry of Public Health, Nonthaburi, Thailand

**Keywords:** Tuberculosis, Migrant, Quality of life, SF-36

## Abstract

**Background:**

Migrants are known to be predominantly poor population which are predisposing to social and health problems, particularly infectious diseases including tuberculosis (TB). TB itself and effect of treatment may further result in substantial morbidity and lowering the quality of life. This study aimed to assess the changes in health-related quality of life (HRQOL) within six months of anti-TB treatment initiation, and the associated factors in Myanmar migrants under anti-TB treatment within this border area.

**Methods:**

This was a prospective cohort study of adult Myanmar migrants with new TB who were within two months of treatment initiation in two TB clinics in Mae Sot, a Thai-Myanmar border area between September 2019 and July 2020. Eight individual domain scores of the HRQOL and Physical and Mental Component Summary (PCS and MCS) scores measured by SF-36 were calculated at month-2 (T1) as baseline, and at the month-4 (T2) and month-6 follow-up visits (T3). Generalized estimation equation models were used to assess the longitudinal changes in PCS and MCS scores of HRQOL.

**Results:**

Of the 155 patients recruited, 93 (60.0%) and 65 (69.9%) completed the month-4 and month-6 follow-ups, respectively. Both the PCS (+ 6.1) and MCS (+ 6.3) scores significantly improved between T1 and T3, with the lowest scores being general health, with the least improvement in social function (+ 1.5) compared with the other domains. Migrants with ethnic origin of Burmese or other were associated with higher PCS and MCS. Those living with family and having higher numbers of initial TB symptoms were associated with lower PCS and MCS scores. Those diagnosed during routine medical checkup were positively associated with PCS scores, whereas patients diagnosed during active case findings were negatively associated with MCS scores. Patients who received residential TB care had higher PCS scores than those with OPD-based TB care.

**Conclusions:**

Continuous improvement in quality of life was found among Myanmar migrants with TB during treatment but their quality of life is still low. Patients with low mental health, especially in the social domain, requires further attention. Active screening policy and supportive strategies during treatment are essential to TB migrants.

## Background

Tuberculosis (TB) remains a major public health problem worldwide. Over 10 million people contract TB and there are 1.3 million TB deaths annually, of which 45% occur in South-East Asia [1]. Active TB has a substantially detrimental impact on health-related quality of life (HRQOL) in both physical well-being and psychological distress or depression [2, 3]. Integrated patient’s perspectives and quality of life should therefore be regarded as essential clinical and outcome markers of anti-TB treatment [4–6]. Previous studies have produced varying findings on HRQOL in TB patients under treatment, from improving drastically within the intensive period to slowing down afterwards [2, 3, 7–9].

Migrants are predisposed to contracting TB [10], which may be related to their social determinants of health that carries a risk of health and/or social well-being problems [11, 12]. A combination of TB infection and migration status poses a higher risk to worsen HRQOL, and notably increases their recovery time compared to the general TB population in the host countries. To date, only a few longitudinal studies in Asian populations have explored HRQOL during anti-TB treatment [13–15]. There have been two studies examining HRQOL among internal migrants in China [16, 17], and a further study from Canada, in 2015, reporting a comparative analysis of HRQOL between active and latent TB infections among a 90% foreign-born population [18]. However, the regions of origin were mostly from Africa, Europe, and America.

To realize the aim of the Sustainable Development Goals on eliminating TB by 2035, TB screening and treatment should be systematically applied in all high-risk regions for all residents, including low-socioeconomic migrants, so as not to leave migrants behind. As migration across international borders has become increasingly frequent during recent decades, proper TB prevention and control is vital in border areas that receive a large number of migrants [19]. However, there has been no longitudinal study on HRQOL among migrants with TB. Mae Sot, Thailand, is an economically important border area between Thailand and Myanmar, with a substantial number of Myanmar migrants crossing every year. A recent study examined available non-profit organizations helping migrants with TB to overcome the barriers of TB care in this border area [20]. Hence, this study aimed to assess the changes in health-related quality of life (HRQOL) within six months of anti-TB treatment initiation, and the associated factors in Myanmar migrants under anti-TB treatment within this border area.

## Methods

### Study design and setting

This is part of an ongoing cohort study conducted in Tak province, a border area of Thailand and Myanmar which are neighboring countries in the South-East Asia region. In this area, there are two TB clinics, Mae Sot District Hospital and Shoklo Malaria Research Unit (SMRU), providing care for more than 200 new TB cases annually. The Mae Sot District Hospital collaborates with the Thai Health Authority as well as various non-profit organizations within the region to provide TB care and treatment for migrants. Since 2009, the SMRU has provided a TB program for migrants living in the border area including TB treatment, residential care, food, psycho-social counselling and other necessities free of charge.

### Study sample

All Myanmar migrants, aged 18 years or above, with new TB who were under two months of anti-TB treatment at the study clinics between 1^st^ September 2019 and 31^st^ July 2020, were included in this study. Multi-drug resistant patients, prisoners, or critically ill cases were excluded from the study. Using the formula of two-dependent means difference considering quality of life scores from a previous study [21], we estimated the means difference of 5 points between two measurements, with a standard deviation of 10, 95% confidence interval and a power of 80%. A final sample size of 64 migrants with TB and having completed 6 months of treatment was required.

### Study measurements

A structured questionnaire, including the details of socio-demographic and clinical characteristics and the 36-Item Short Form Health Survey Version 2 (SF-36v2) for HRQOL, was used. Sociodemographic and clinical characteristics were recorded at baseline time point within 2 months of treatment (T1). HRQOL was measured at three time points: at T1, at 4-months of treatment (T2), and at 6-months of treatment (T3), respectively.

Socio-demographic characteristics consisting of age, gender, ethnicity, literate, marital status, place of residence, having a job at TB diagnosis, wage type, type of household, documentation status, and health insurance status, were obtained from patient interviews. Their ability to understand the local Thai language was also noted. Clinical characteristics, including number of initial TB symptoms (persistent cough for 2 or more weeks, sputum, hemoptysis, chest pain, weight loss, loss of appetite, fever, or night sweats), type of healthcare sought, type of TB, chest X-Ray (CXR) findings, smear results, Gene X-pert results, human immunodeficiency virus (HIV) status, and type of care, were collected from the patients’ medical records. Residential care was defined as care provided together with shelter, food, anti-TB treatment and other essentials for at least two months.

The validated and widely used SF-36v2 questionnaire was used for gathering HRQOL information [2, 4]. The SF-36v2 questionnaire contains 36 questions and generates scores for 8 domains of HRQOL including physical functioning (10 items), role limitations due to physical problems (4 items), bodily pain (2 items), general health (5 items), vitality (4 items), emotional well-being (5 items), role limitations due to emotional problems (3 items), and social functioning (2 items) [22]. These 8 domain scores were translated into norm-based scores, with a mean of 50 and standard deviation of 10. These scores were then used to construct physical (PCS) and mental component scores (MCS), by multiplying each score with weights obtained from the oblique factor solution method. The final summary components were scored out of 100 and higher scores indicate better HRQOL [23].

The questionnaire in English was finalized after modifications into local context conducted with the help of TB experts working within the border area. It was then translated into the Burmese language, and then translated back into English by another independent translator to check the linguistic validity of translation. This was then pilot tested among 9 TB migrants that were not included in the study. Internal consistency was measured using Cronbach’s alpha, and the value was 0.89.

### Data collection

In order to minimize interview bias, two Karen research assistants, who spoke both Burmese and Karen and had previous experience working in the health sector, but not in participating clinics, were recruited and trained prior to the study. On the clinic day, the clinic nurse introduced eligible TB migrants to the research team. Patients were then informed about the study, in either their mother tongue or in a language they understood. After obtaining consent from the patient the researchers conducted the initial interview (T1). At the end of the interview day, the researchers checked the completeness of questionnaires on a daily basis. At the appropriate follow-ups, the patients would then undergo the second (T2) and third (T3) interviews.

### Statistical analysis

Data were entered into EpiData Manager and Entry-Client Software version 4.6.0.2, and analyzed using R version 3.6.2 (The R Foundation for Statistical Computing, Vienna, Austria, 2019). Patients’ socio-demographic and clinical characteristics were described using descriptive statistics. The changes of HRQOL at each time point (T2-T1, T3-T2 and T3-T1), for each domain, and summary scores on PCS and MCS were analyzed using paired t-tests. The longitudinal changes of PCS and MCS were also analyzed using Generalized Estimating Equation (GEE) models, with an autoregressive correlation structure [24]. All socio-demographic and clinical characteristics were put in the GEE model using a backward stepwise method. A *P*-value less than 0.05 was regarded as significant.

## Results

**Fig. 1:**
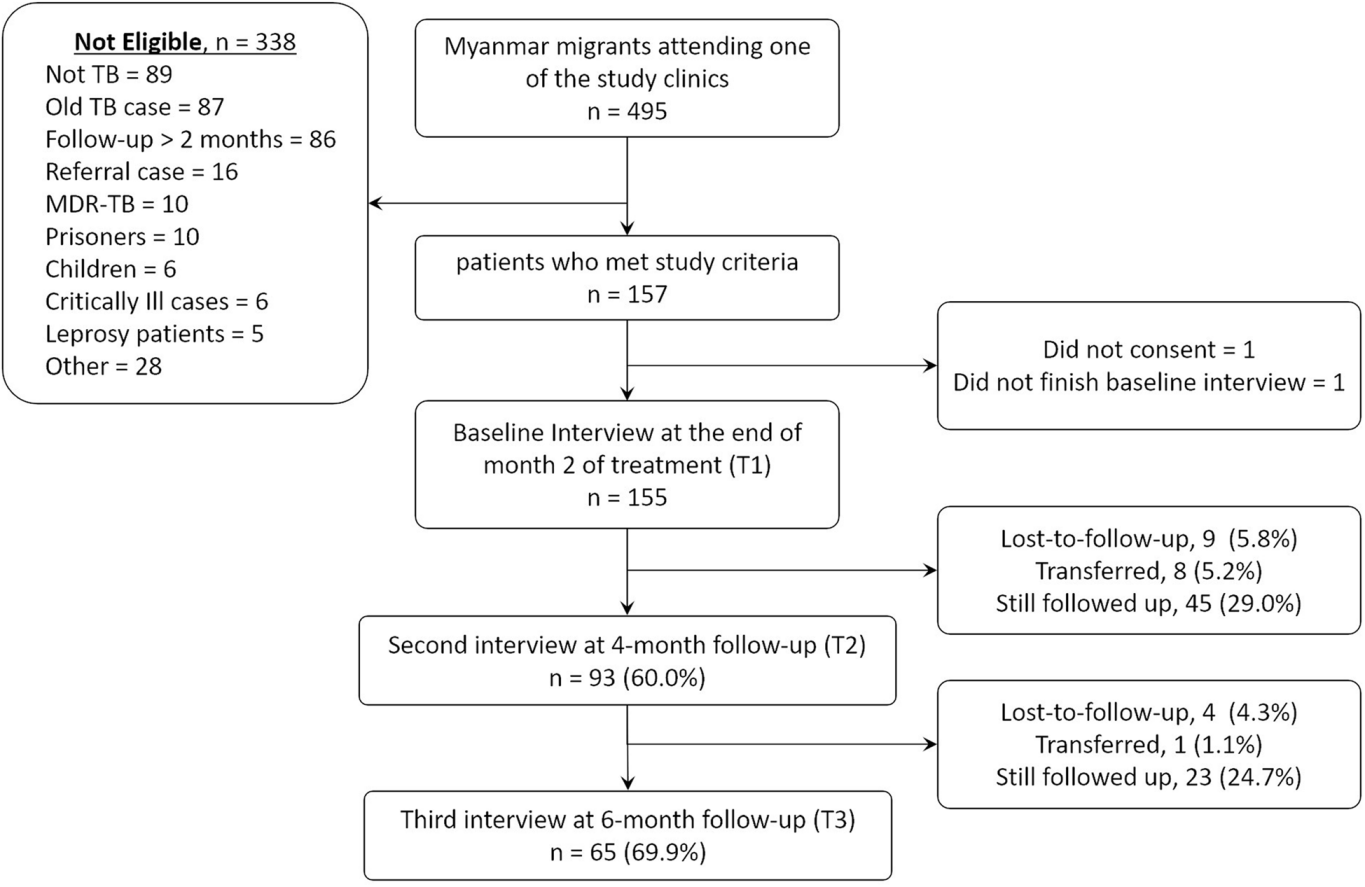
Flow diagram of the study. TB, tuberculosis; MDR-TB, multi-drug resistant tuberculosis. “Other” refers to death, receiving treatment at another health facility close to their residence or receiving treatment at their residence

From 155 patients recruited at T1, 65 patients completed the 6-month follow-up, 68 patients were still being followed up, 9 had been transferred, and 13 had been lost-to-follow-up. Figure 1 shows the flow diagram of the study. The socio-demographic and clinical characteristics of all study patients are presented in Tables 1 and 2, respectively. The patients were predominantly less than 45 years of age, male, Burmese, literate, married, had a job at the time of TB diagnosis, and resided within Mae Sot. Only one-fourth earned a regular monthly salary. Almost half of them had no legal documentation, and two-thirds were not insured at the time of TB diagnosis.

**Table 1:** Socio-demographic characteristics of patients at baseline visit (*n* = 155)

Variable	Category	Baseline n (%)
Age in years	18–35	66 (42.6)
	35–44	41 (26.5)
	45+	48 (31.0)
Gender	Male	101 (65.2)
	Female	54 (34.8)
Ethnicity	Burmese	96 (61.9)
	Karen	37 (23.9)
	Mon	4 (2.6)
	Other^a^	18 (11.6)
Literate	Yes	122 (78.7)
	No	33 (21.3)
Marital Status	Married	94 (60.6)
	Not Married	61 (39.4)
Place of residence	Within Mae Sot	129 (83.2)
	Outside Mae Sot	26 (16.8)
Having a job at TB diagnosis	Yes	129 (83.2)
	No	26 (16.8)
Wage type	Daily	62 (40.0)
	Monthly	40 (25.8)
	Other	27 (17.4)
	Missing	26 (16.8)
Type of household	Living alone	29 (18.7)
	Living with family	113 (72.9)
	Living with others	13 (8.4)
Documented migrant	Yes	90 (58.1)
	No	65 (41.9)
Health insurance	Yes	54 (34.8)
	No	101 (65.2)
Thai language	Know a few words	92 (59.4)
	Do not understand at all	41 (26.5)
	Can speak well	22 (14.2)

*TB* tuberculosis

^a^ Other refers to Rakhine, Chinese, Naga, Pa Oh and Gaw Rakhar

**Table 2:** Clinical characteristics of patients at baseline visit (n = 155)

Variable	Category	Baseline n (%)
Number of Initial TB Symptoms	0	22 (14.2)
	1	19 (12.3)
	2	33 (21.3)
	3	29 (18.7)
	4+	52 (33.5)
Type of healthcare sought	Passive case finding	126 (81.3)
	Active case finding	9 (5.8)
	Medical Checkup	20 (12.9)
Type of TB	Pulmonary TB	115 (74.2)
	Extra-Pulmonary TB	14 (9.0)
	Missing	26 (16.8)
Chest X-Ray findings	Normal	19 (12.3)
	Suggestive of TB	92 (59.4)
	Suggestive of Other	10 (6.5)
	Missing	34 (21.9)
Smear result	1+	26 (16.8)
	2+	15 (9.7)
	3+	30 (19.4)
	Negative	53 (34.2)
	Not Done	5 (3.2)
	Missing	26 (16.8)
Gene X-pert result	MTB Detected	58 (37.4)
	MTB Not Detected	27 (17.4)
	Not Done	44 (28.4)
	Missing	26 (16.8)
HIV Status	Negative	102 (65.8)
	Known Case	5 (3.2)
	Newly Diagnosed	9 (5.8)
	Missing	26 (16.8)
Type of care	OPD-based	94 (60.6)
	Residential	61 (39.4)

*TB* tuberculosis; *MTB Mycobacterium tuberculosis*; *OPD* outpatient department

Table 3 shows the differences in mean HRQOL scores between the different measurements. Significant mean score differences at T2-T1 and T3-T1 were found in most domains as well as the summary components, except in pain and social functioning. The mean MCS scores were consistently lower than the PCS scores. Large improvements of both PCS (+ 4.7) and MCS (+ 6.0) at T2-T1 were observed, but the trend slowed down for PCS (+ 1.5) and plateaued for MCS (+ 0.2) at T3-T2.

**Table 3:** Differences in mean HRQOL SF-36 scores between T2-T1, T3-T2 and T3-T1

Scale	Items	Baseline T1 n = 155 Mean (SD)	T2 - T1 *n* = 93	T3 - T2 *n* = 65	T3 - T1 n = 65
Physical functioning	10	48.6 (11.7)	+ 3.3 *	+ 0.6	+ 4.0 **
Role functioning/physical	4	46.5 (11.1)	+ 6.1 ***	+ 0.6	+ 8.0 ***
Role functioning/emotional	3	45.8 (12.3)	+ 6.6 ***	+ 0.2	+ 7.6 ***
Vitality	4	53.3 (11.7)	+ 5.4 ***	+ 0.0	+ 5.3 **
Mental health	5	47.5 (12.2)	+ 6.7 ***	− 0.5	+ 6.4 **
Social functioning	2	55.1 (6.4)	+ 1.1	+ 1.0	+ 1.5
Pain	2	52.1 (9.8)	+ 2.5 *	+ 2.4 *	+ 4.5 **
General health	5	42.4 (8.0)	+ 2.9 **	+ 1.0	+ 4.5 ***
Physical component score	21	57.5 (8.2)	+ 4.7 ***	+ 1.5 *	+ 6.1 ***
Mental component score	14	55.7 (9.4)	+ 6.0 ***	+ 0.2	+ 6.3 ***

*HRQOL* health-related quality of life; *SF-36* Short-form 36; *T1* measurement at the end of month 2; *T2* measurement at the end of month 4; *T3* measurement at the end of month 6; *SD* standard deviation

* p-value < 0.05, ** p-value < 0.01, *** *p*-value < 0.001 (using paired t-tests)

The factors influencing changes in the PCS and MCS scores of HRQOL in the GEE models are presented in Table 4. Significant increasing trends of both PCS and MCS scores were detected over the follow-up visits, and in patients with ethnic origins of Burmese or other, of which the majority were Rakhine. Those living with family and having higher numbers of initial TB symptoms were associated with lower PCS and MCS scores. Those diagnosed during routine medical checkups were positively associated with PCS scores; whereas, patients diagnosed during active case findings were negatively associated with MCS scores. Patients who received residential TB care had higher PCS scores than those with OPD-based TB care.

**Table 4:** Generalized Estimating Equation (GEE) models for factors influencing changes in physical and mental health component scores (n = 65)

Category	Sub-category	PCS			MCS		
		β	SE	p-value	β	SE	p-value
Intercept		**56.8**	**2.1**	**< 0.001**	**60.2**	**2.8**	**< 0.001**
Visit	T1	Ref			Ref		
	T2	**4.6**	**1.1**	**< 0.001**	**6.1**	**1.3**	**< 0.001**
	T3	**6.1**	**1.2**	**< 0.001**	**6.3**	**1.5**	**< 0.001**
Ethnicity	Karen	Ref			Ref		
	Burmese	**3.0**	**1.3**	**0.020**	**4.0**	**1.5**	**0.007**
	Mon	1.0	4.1	0.816	3.2	2.6	0.222
	Other^a^	**4.0**	**1.6**	**0.014**	**5.7**	**1.9**	**0.003**
Type of household	Living alone	Ref			Ref		
	Living with family	**−2.9**	**1.1**	**0.007**	**−3.7**	**1.4**	**0.007**
	Living with others	1.8	1.7	0.297	2.1	2.2	0.346
Number of initial TB Symptoms	0	Ref			Ref		
	1	**−5.5**	**1.9**	**0.004**	**−8.9**	**2.2**	**< 0.001**
	2	−2.9	1.6	0.081	**−7.3**	**2.3**	**0.001**
	3	−1.9	1.5	0.202	**−4.9**	**2.1**	**0.016**
	4+	−1.5	1.5	0.310	**−6.4**	**2.1**	**0.003**
Type of healthcare sought	Passive case finding	Ref			Ref		
	Active case finding	−1.0	2.0	0.623	**− 6.4**	**2.7**	**0.017**
	Medical Checkup	**3.2**	**1.5**	**0.038**	0.9	2.0	0.664
Type of TB care	OPD	Ref			–	–	–
	Residential	**2.5**	**1.2**	**0.032**	–	–	–

*PCS* physical component score; *MCS* mental component score; *β* beta coefficient; *SE* standard error; *T1* measurement at the end of month 2; *T2* measurement at the end of month 4; *T3* measurement at the end of month 6; *OPD* out-patient department

^a^ Other refers to Rakhine, Chinese, Naga, Pa Oh and Gaw Rakhar

## Discussion

The majority of HRQOL domains among the study’s Myanmar migrants with TB showed significant improvements over time, up until 6 months of treatment, in this longitudinal analysis. The PCS scores continuously improved throughout the study, while the MCS scores increased significantly at 4 months of treatment, and then plateaued during the remaining follow-up period. The poorest results were found in the social functioning domain. Ethnicity, type of household, higher number of initial TB symptoms, type of healthcare sought, and type of TB care were predictors for changes in the HRQOL.

Clinically significant improvements in most HRQOL domains, and component scores between T1 and T3 found in our longitudinal study were better than the findings from three previous studies conducted among general populations with active TB. These being a study from Uganda and two studies from Canada [3, 18, 25]. This difference may be explained by different baseline clinical conditions of the Myanmar migrants along with the healthcare system set up for TB control at the Thailand-Myanmar border [26]. A systematic review of HRQOL studies in TB patients found a heterogeneity of studies in terms of patient characteristics, HRQOL measures, time points of assessments, and study design [2].

Significantly increasing trends in both PCS and MCS between T1 and T2, followed by a slowdown in PCS, with little change in MCS afterwards to T3 were found in our study. These were different from a study conducted in Canada which showed little or no improvement in PCS, but a decline in MCS trends through months 2, 4 and 6 of follow-up [18]. In addition, these findings could be the result of the lowest score being within the general health domain among the migrant patients at the beginning. A systematic review on HRQOL also reported the largest improvements were between treatment initiation and 2–3 months of treatment [2, 15]. This may be due to different characteristics in the study participants, study design; this being either longitudinal or cross-sectional, and the different times of assessments.

Little improvement seen over the study period in the social functioning domain among our migrant population is similar to the findings from a previous study conducted in the general TB population of China [4], but lower than the findings from a study among foreign-born TB patients in Canada [3]. The explanation for this could be that the migration status in our group predisposed them to the feelings of stigma, low HRQOL and mental health problems [27, 28]. Additionally, these factors could be worsened due to their disease, even when undergoing treatment.

Our longitudinal analysis confirmed the improvement of PCS and MCS scores over time after adjusting for other independent factors. This improvement on the mental health component of HRQOL was supported by the summary of a scoping review, which reported that the most noticeable effect on the mental health of TB patients occurred between diagnosis and a few months after treatment initiation, and then some improvement afterwards [29]. In our study, Burmese people and other ethnic groups were found to have better HRQOL scores over time compared to the Karen migrants. This may be explained by the findings from a qualitative study in the same border area showing that Karen patients had different health beliefs, and tolerance to illness compared to other ethnic groups that might affect their health-seeking behaviors, and reduce their quality of life [30].

We could not find the previous study to support our finding on lower HRQOL scores among TB migrants who lived with family members. This could be assumed that TB can impose a disproportionate financial burden on poor households [31] as they may not be able to work as usual. Furthermore, TB migrants have to isolate him/herself from the family to prevent the transmission of the diseases, potentially causing stigma and discrimination within the household. These events can trigger psychological stress and depression in TB patients [32], contributing to lower HRQOL scores. In addition, higher numbers of initial TB symptoms are associated with severe conditions of the disease at the time of diagnosis, affecting the quality of life [33]. Although migrants with active TB discovered during a routine medical checkup may show no or fewer symptoms, their quality of life may benefit from early diagnosis. Residential care TB migrants receiving support for their basic needs as well as those being part of a community may mitigate the extent of social exclusion. Additionally, this may help with their medication adherence, reinforce the links with family members and health professionals, and subsequently improving their quality of life [34].

From our literature search, there were no longitudinal studies measuring HRQOL among migrants under anti-TB treatment. Our study setting was in a landlocked border area, between Myanmar and Thailand, and involving two large clinics treating migrants with TB. There are a few limitations to our study. First, we excluded multi-drug resistant TB cases, prisoners, and very ill cases; which could have led to overestimation of HRQOL scores. Second, we analyzed the data from 65 migrants who completed the three HRQOL assessments: this number was consistent with the sample size calculated. Migrants who were lost-to-follow-up, or transferred may have produced attrition bias. However, we explored their baseline characteristics and HRQOL, compared with those who completed measurements, and found there was no statistical significance; indicating small bias. Third, we focused only one group of migrants with TB under their treatment without a comparable group of migrants which may influence the migrant’s response to HRQOL due to Hawthorne effect. Finally, we examined Myanmar migrants in a specific border area, thus the generalizability may be limited for migrants from other countries, or in other border areas of Thailand.

Our results highlight the low HRQOL, particularly in mental aspect among migrants with TB, even though it improved. These findings indicated the need for targeted and migrant-sensitive psychosocial support interventions to improve their HRQOL. Annual health checkups, regardless of symptoms for early diagnosis and close intensive treatment, with optional residential care that could provide physical and mental support should be integrated in the Thai health system and related policies for migrants.

## Conclusion

Good improvement at the end of 4-months of treatment, and then a plateauing in HRQOL scores was found among Myanmar migrants with TB in a border area of Myanmar and Thailand. Their mental health, particularly in the social functioning domain, was poorer than their physical health. Social, clinical and treatment factors are important to HRQOL improvement.

## Data Availability

The datasets used and/or analyzed during the current study are available from the corresponding author on reasonable request.

## Abbreviations

CXR: Chest X-Ray
GEE: Generalized Estimating Eq.
HIV: Human immunodeficiency virus
HRQOL: Health-related quality of life
MCS: Mental Component Summary
PCS: Physical Component Summary
SF-36v2: Short Form Health Survey Version 2
SMRU: Shoklo Malaria Research Unit
OPD: Out-patient department
TB: Tuberculosis
